# Antimicrobial Activity of Colistin Against Contemporary (2015 – 2017) *P. aeruginosa* and *A. baumannii* Isolates From a Chinese Surveillance Program

**DOI:** 10.3389/fmicb.2020.01966

**Published:** 2020-09-04

**Authors:** Hui Zhang, Ge Zhang, Jingjia Zhang, Simeng Duan, Yue Kang, Qiwen Yang, Yingchun Xu

**Affiliations:** ^1^Department of Clinical Laboratory, Peking Union Medical College Hospital, Peking Union Medical College, Chinese Academy of Medical Sciences, Beijing, China; ^2^MRL Global Medical Affairs, MSD China, Shanghai, China

**Keywords:** *Pseudomonas aeruginosa*, *Acinetobacter baumannii*, carbapenem, colistin, RTI, IAI, UTI

## Abstract

**Objective:**

To investigate the incidence and susceptibilities of non-fermenting bacteria isolates from Chinese respiratory (RTI), intra-abdominal (IAI) and urinary tract (UTI) infections to antimicrobial agents between 2015 and 2017.

**Methods:**

In total, 3,246 non-fermentative bacteria were collected from 21 hospitals and 9 hospital departments across 7 regions of China. A central testing laboratory was employed to determine antimicrobial susceptibilities using appropriate standards of interpretation.

**Results:**

The majority of the isolates were *Acinetobacter baumannii* (*n* = 1,360, 41.9%) and *Pseudomonas aeruginosa* (*n* = 1,341, 41.3%). Overall multidrug resistance (MDR) and carbapenem resistance (CR) rates of *Acinetobacter baumannii* were 80.1 and 78.7% with MDR and CR rates in RTIs, IAIs, and UTIs of 82.0 and 81.0%, 82.6 and 81.0% as well as 53.1 and 46.9%. Overall MDR and CR rates of *Pseudomonas aeruginosa* isolates were 36.2 and 38.9% with 41.8 and 44.3%, 29.3 and 36.1% as well as 24.2 and 20.2% MDR and CR rates in RTIs, IAIs, and UTIs. Overall susceptibility rates to imipenem, meropenem, amikacin, ciprofloxacin, cefepime and piperacillin-tazobactam were 21.1, 21.3, 33.0, 18.4, 19.2, and 19.6% for *Acinetobacter baumannii* and 56.5, 58.5, 88.4, 63.1, 63.1, and 55.63% for *Pseudomonas aeruginosa* isolates, whereas for colistin they were 95.7 and 94.6%, respectively. In all departments and regions of China, susceptibility rates of *Pseudomonas aeruginosa* and *Acinetobacter baumannii* isolates to colistin were constantly above 80%.

**Conclusion:**

Due to the high MDR and CR rates for *Pseudomonas aeruginosa* and *Acinetobacter baumannii*, isolates obtained from RTIs, IAIs, and UTIs only maintained high susceptibility rates to colistin between 2015 and 2017.

## Introduction

Non-fermenting bacteria are a group of aerobic or facultative anaerobic, spore-free, Gram-negative bacteria that do not use glucose or use only oxidized forms; most are conditional pathogens, which mainly cause nosocomial infections, and clinical treatment is very difficult (Mesaros et al., 2007; Perez et al., 2007). In recent years, due to the extensive application of various medical invasive examinations, diagnosis and treatment techniques, as well as broad-spectrum antibacterial drugs, the number of nosocomial infections caused by non-fermentation bacteria has gradually increased, with *Acinetobacter baumannii* (*A. baumannii*) and *Pseudomonas aeruginosa* (*P. aeruginosa*) being the most common.

*Acinetobacter baumannii* is an opportunistic pathogen and is one of the most common nosocomial pathogens in clinical practice in China (Chen et al., 2012). Due to the widespread use of antibacterial drugs, especially β-lactam antibiotics, the isolation rates of *A. baumannii* in clinical trials are on the rise, and its drug resistance has also shown an upward trend. Indeed, multi-drug resistant and even pan-resistant strains are common (Chopra et al., 2013). *A. baumannii* often causes respiratory, urinary, skin, soft tissue and bloodstream infections. It is one of the main pathogens causing ventilator-associated pneumonia in the United States and Europe (Fernández-Barat et al., 2017; Koulenti et al., 2017). The pathogenicity and drug resistance mechanism of *P. aeruginosa* have recently been extensively investigated (Dimopoulos et al., 2020). An investigation into healthcare associated infections in the US revealed that *P. aeruginosa* was the 6th most commonly found pathogen in infections as well as the 2nd most common organism causing pneumonia (Magill et al., 2014). Furthermore, this organism has evolved various defenses that render it resistance to various classes of antibiotics including the β-lactams; *P. aeruginosa* is often cited as being multidrug resistant (MDR) (Castanheira et al., 2014). This has serious consequences as antibiotic therapy to treat MDR *P. aeruginosa* are severely limited, particularly in areas of China where MDR organisms are widely dispersed (Ruiz-Garbajosa and Canton, 2017; El Chakhtoura et al., 2018).

As MDR *P. aeruginosa* and *A. baumannii* infections are increasing, available antibiotics to treat infections are limited (Karageorgopoulos and Falagas, 2008; Pachon and Vila, 2009; Towner, 2009). Colistin may be one of the most effective antibiotics against *P. aeruginosa* or *A. baumannii* resistant to carbapenems (Yuan and Tam, 2008), but as a last line of defense the colistin sensitivities of *P. aeruginosa* and *A. baumannii* is a major concern. The Study for Monitoring Antimicrobial Resistance Trends (SMART) is a global surveillance project designed to collect and monitor *in vitro* antimicrobial susceptibilities of isolates obtained from patients with intra-abdominal infections (IAIs), respiratory tract infection (RTIs) and urinary tract infections (UTIs).

The present study is the first to investigate and analyze the rate of non-fermenting bacteria isolates in IAIs, UTIs and RTIs, as well as the rates of MDR and carbapenem resistance (CR) *A. baumannii* and *P. aeruginosa* isolates and their sensitivities to colistin using data collected by SMART China from 2015 to 2017.

## Materials and Methods

### Non-fermenting Isolates Obtained From 7 Regions of China (2015 – 2017)

Our hospital’s Human Research Ethics Committee accepted the protocols for the present inquiry (Et Number: S-K238) and waived the requirement for consent.

In total, 3,246 non-fermentative bacteria (2015, *n* = 2,203; 2016, *n* = 2,375; 2017, *n* = 2,535) isolates were collected in 7 distinct regions of China from 21 hospitals (northeast, north, central south, southwest, east Jiangzhe, and east non-Jiangzhe areas) between 2015 and 2017 inclusively. The total number of samples collected yearly from each hospital ranged between 77 and 250. Each sample was identified and dispatched for analysis in Peking Union Medical College Hospital; re-identification employed MALDITOF MS as the main tool. Duplicate isolates from the same patient were not included in the analysis. The detailed collection criteria for isolates from IAI, UTI, and RTI are as follows:

### Sample Collection Criteria for IAIs

The isolate must meet the laboratory criteria of “significant pathogen” and be considered the probable causative agent of infection. Only Gram-negative aerobic and facultative anaerobic bacteria from abdominal infection sites such as the appendix, peritoneum, colon, bile, pelvis and pancreas were included and the strains needed to be pathogenic bacteria associated with clinical infections, while Gram-positive and anaerobic bacteria were excluded. The specimens were mainly obtained through surgical procedures, but puncture specimens such as intraperitoneal puncture fluid were also included and different Gram-negative bacteria that were combined in one sample were also accepted. Exclusion criteria were isolates from drainage liquid or drainage bottles, as well as isolates from feces or perianal abscess environmental samples (not a patient source) or cultures for infection control purposes.

### Sample Collection Criteria for UTIs

The isolate must meet the laboratory criteria of a “significant pathogen” and be considered the probable causative agent of infection. The UTI isolates were obtained from clean catch midstream urine, the urinary bladder, kidney, and the prostate gland.

### Sample Collection Criteria for RTIs

The isolate must meet the laboratory criteria of a “significant pathogen” and be considered the probable causative agent of infection. Gram-negative aerobic and facultative bacteria cultured from specimens from lower respiratory tract body sites [e.g., sputum, bronchoalveolar lavage (BAL), thoracentesis, bronchial brushing, endotracheal aspirate, and lung biopsy].

### Testing of Organism Susceptibility to Antibiotics

Antimicrobial susceptibility testing was performed according to the Clinical and Laboratory Standards Institute (CLSI) broth microdilution method with custom-made dehydrated Trek Diagnostic Systems panels (Thermo Fisher Scientific, Cleveland, United States) between 2015 and 2017. The susceptibility interpretations were based on the clinical breakpoints recommended by the Clinical and Laboratory Standards Institute [CLSI] (2017). The antimicrobial agents colistin, carbapenems (imipenem, meropenem), an aminoglycoside (amikacin), a cephalosporin (cefepime), a quinolone (ciprofloxacin) and piperacillin/azobactam were tested according to the guidelines of the Surgical Infection Society and Infectious Diseases Society of America (Solomkin et al., 2010). Reference strains of *E. coli* American Type Culture Collection (ATCC) 25922 and *P. aeruginosa* (ATCC 27853) were used as quality controls for each batch of MIC tests. Data were analyzed only when the quality control test results were acceptable. In the present study, a CR strain was defined as resistant to either imipenem or meropenem. MDR was defined as a strain that was not susceptibility to ≥3 of 6 key antibiotics namely: amikacin; ciprofloxacin; cefepime; colistin; imipenem; and piperacillin/tazobactam. These antibiotics were chosen as they are usually employed in the clinic to treat Gram-negative infections.

## Results

### Composition of Non-fermenting Bacteria From 2015 – 2017

From 2015 to 2017, a total of 3,246 non-fermentative bacteria were collected, of which *P. aeruginosa* and *A. baumannii* were major isolates of non-fermentative bacteria with similar proportions (41.3 and 41.9%, respectively), with the majority isolated from RTIs. However, the overall percentages of MDR and CR in *A. baumannii* isolates were as high as 80.1 and 78.7%, respectively, which was almost two times that of MDR *P. aeruginosa* (36.2%) and CR *P. aeruginosa* (38.9%) rates. *A. baumannii*, CR and MDR phenotypes were particularly found in abundance in isolates taken from the respiratory tract (81.0 and 82.0%) and in IAIs (81.0 and 82.6%). Similarly, MDR and CR rates of *P. aeruginosa* strains were essentially highest in isolates from RTIs (41.8 and 44.3%) followed by IAI isolates (29.3 and 36.1%) and lowest in UTI isolates (24.2 and 20.2%). Apart from somewhat lower MDR *P. aeruginosa* and CR *P. aeruginosa* rates in 2015 there was otherwise an almost constant pattern throughout the years 2015–2017 (Table 1).

**TABLE 1:** The overall distribution of non-fermentative bacteria, MDR non-fermentative bacteria and CR non-fermentative bacteria collected in 2015, 2016, and 2017.

**2015-2017**	**No. isolated N (%)**	**MDR N (%)**	**CR N (%)**
Non-fermentative bacteria	3246 (100.0)	1754 (54.0)	1699 (52.3)
*A. baumannii*	1360 (41.9)	1090 (80.1)	1070 (78.7)
IAI	327 (24.0)	270 (82.6)	265 (81.0)
UTI	96 (7.1)	51 (53.1)	45 (46.9)
RTI	933 (68.6)	765 (82.0)	756 (81.0)
*P. aeruginosa*	1341 (41.3)	486 (36.2)	522 (38.9)
IAI	321 (23.9)	94 (29.3)	116 (36.1)
UTI	198 (14.8)	48 (24.2)	40 (20.2)
RTI	815 (60.8)	341 (41.8)	361 (44.3)
Other non-fermentative bacteria	545 (16.8)	178 (32.7)	107 (19.6)
**2015**			
Non-fermentative bacteria	742	380 (51.2)	363 (48.9)
*A. baumannii*	283 (38.1)	230 (81.3)	224 (79.2)
IAI	115 (40.6)	98 (85.2)	97 (84.3)
UTI	25(8.8)	14(56.0)	10 (40.0)
RTI	141 (49.8)	116(82.3)	115 (81.6)
*P. aeruginosa*	320 (43.1)	88 (27.5)	104 (32.5)
IAI	118 (36.9)	32 (27.1)	42 (35.6)
UTI	53 (16.6)	4 (7.5)	4 (7.5)
RTI	149 (46.6)	52(34.9)	58 (38.9)
Other non-fermentative bacteria	139 (18.7)	62 (44.6)	35 (25.2)
**2016**			
Non-fermentative bacteria	1091	616 (56.5)	599 (54.9)
*A. baumannii*	492 (45.1)	393 (79.9)	384 (78.0)
IAI	111 (22.6)	91 (82.0)	87 (78.4)
UTI	37 (7.5)	19 (51.4)	17 (45.9)
RTI	343 (69.7)	282 (82.2)	279 (81.3)
*P. aeruginosa*	449 (41.2)	176 (39.2)	184 (41.0)
IAI	108 (24.1)	37 (34.3)	44 (40.7)
UTI	46 (10.2)	11 (23.9)	10 (21.7)
RTI	290 (64.6)	125 (43.1)	125 (43.1)
Other non-fermentative bacteria	150 (13.7)	47 (31.3)	31 (20.7)
**2017**			
Non-fermentative bacteria	1413	758 (53.6)	737 (52.2)
*A. baumannii*	585 (41.4)	467 (79.8)	462 (79.0)
IAI	101 (17.3)	81 (80.2)	81 (80.2)
UTI	34 (5.8)	18 (52.9)	18 (52.9)
RTI	449 (76.8)	367 (81.7)	362 (80.6)
*P. aeruginosa*	572 (40.5)	222 (38.8)	234 (40.9)
IAI	95 (16.6)	25 (26.3)	30 (31.6)
UTI	99 (17.3)	33 (33.3)	26 (26.3)
RTI	376 (65.7)	164 (43.6)	178 (47.3)
Other non-fermentative bacteria	256 (18.1)	69 (27.0)	41 (16.0)

*Other non-fermentative bacteria include: *Acinetobacter junii; Aeromonas hydrophila; Aeromonas caviae; Acinetobacter lwoffii; Alcaligenes faecalis; Alcaligenes xylosoxidans; Alcaligenes xylosoxidans* ssp*.; Morganella morganii; Pseudomonas putida; Stenotrophomonas maltophilia; Flavobacterium meningosepticum; Burkholderia cepacia; Pseudomonas fluorescens.**

### Susceptibility Analysis of *A. baumannii* and *P. aeruginosa* to 7 Antimicrobial Agents (2015 – 2017)

**FIGURE 1:**
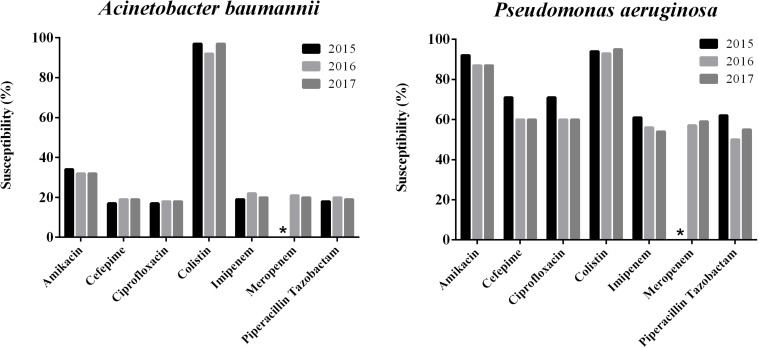
Trends of antimicrobial susceptibilities of *P. aeruginosa* and *A. baumanni* isolates collected from 2015 to 2017 in China. ^∗^Means no data available.

The susceptibility rates to carbapenems of *A. baumannii* isolates were only about 20% and that of *P. aeruginosa* was <60%. Susceptibility rates of *P. aeruginosa* isolates were relatively high to amikacin (88.4%), but the MIC_90_ values were >32 mg/L, while *A. baumannii* isolates exhibited only 33.04% susceptibility. The susceptibility rates to ciprofloxacin, cefepime and piperacillin tazobactam were higher *in P. aeruginosa* isolates (55 – 63%) than in *A. baumannii* isolates (<20%). The susceptibility rates to colistin of both *P. aeruginosa* and *A. baumannii* were >90% (94.6 and 95.7%, respectively) and both MIC_50_/MIC_90_ values were ≤1 mg/L (Table 2). The susceptibilities of both *P. aeruginosa* and *A. baumannii* to colistin has been changing in the shape of a “U” curve over the 3 years of the study (Figure 1).

**TABLE 2:** Susceptibility rates of *A. baumannii* and *P. aeruginosa* to 7 antimicrobial agents.

**Organism/antimicrobial agent**	***N***	**MIC (mg/L)**			**MIC interpretation**		
		**MIC_50_**	**MIC_90_**	**MIC range**	**Susceptible (%)**	**Intermediate (%)**	**Resistant (%)**
***A. baumannii***							
Colistin	1356	≤1	≤1	≤1–>8	95.7	0	4.4
Imipenem	1356	32	>32	≤0.5–>32	21.1	0.7	78.2
Meropenem	1075	>16	>16	≤0.12–>16	21.3	0.7	78.0
Amikacin	1356	>32	>32	≤4–>32	33.0	0.7	66.3
Ciprofloxacin	1356	>2	>2	≤0.25–>2	18.4	0.6	81.0
Cefepime	1356	>32	>32	≤1–>32	19.2	2.3	78.5
Piperacillin Tazobactam	1356	>64	>64	≤2–>64	19.6	1.2	79.2
***P. aeruginosa***							
Colistin	1341	≤1	2	≤1–>8	94.6	0	5.4
Imipenem	1341	2	32	≤0.5–>32	56.5	6.0	37.5
Meropenem	1021	1	>16	≤0.12–>16	58.5	6.5	35.1
Amikacin	1341	≤4	>32	≤4–>32	88.4	0.8	10.8
Ciprofloxacin	1341	0.5	>2	≤0.25–>2	63.1	6.0	31.0
Cefepime	1341	8	>32	≤1–>32	63.1	11.1	25.8
Piperacillin Tazobactam	1341	16	>64	≤2–>64	55.6	13.3	31.1

*According to CLSI Performance Standards for Antimicrobial Susceptibility Testing, 27th Edition in 2017, MIC breakpoints (mg/L) to colistin for *A. baumannii* are ≤2 for susceptibility and >2 for resistance, while MIC breakpoints (mg/L) to colistin for *P. aeruginosa* are ≤2 for susceptibility and ≥4 for resistance.*

### Susceptibility Analysis of MDR *A. baumannii*, CR *A. baumannii*, MDR *P. aeruginosa* and CR *P. aeruginosa* to Colistin for Different Infection Types From 2015 to 2017

Colistin (96.1 and 95.4%) demonstrated highest susceptibility in CR *A. baumannii* and MDR *A. baumannii* isolates while susceptibilities to the other antimicrobial agents, with the exception of amikacin (16.8%), including imipenem as well as ciprofloxacin were <3%. Colistin susceptibility rates of CR and MDR *A. baumannii* isolates were 95.4 and 95.4% for respiratory tract 98.1 and 97.0% for IAIs, as well as 95.6 and 92.2% for UTIs. Susceptibility rates of CR and MDR *A. baumannii* isolates to amikacin were in the range of 17 – 18% for RTI, 14 – 15% for IAIs and 7 – 9% for UTIs, whereas with the exception of MDR *A. baumannii* from UTIs with susceptibility rates of 2 – 12%, all CR and MDR *A. baumannii* isolates from RTIs, IAIs and UTIs exhibited < 3% susceptibilities to all other tested antimicrobial agents (Table 3).

**TABLE 3:** *In vitro* activity of 7 antimicrobial agents against CR and MDR *A. baumannii* collected in China from 2015 to 2017.

**Organism/specimen source/antimicrobial agent**	***N***	**MIC (mg/L)**			**MIC interpretation**		
		**MIC_50_**	**MIC_90_**	**MIC range**	**Susceptible (%)**	**Intermediate (%)**	**Resistant (%)**
**CR-*A. baumannii*, all sources**							
Colistin	1070	≤1	≤1	≤1 to >8	96.1	0	3.9
Amikacin	1070	>32	>32	≤4 to >32	16.8	0.8	82.3
Cefepime	1070	>32	>32	2 to >32	1.3	2.1	96.6
Ciprofloxacin	1070	>2	>2	≤0.25 to >2	1.8	0.6	97.7
Imipenem	1070	32	>32	1 to >32	0.4	0.1	99.5
Meropenem^∗^	846	>16	>16	1 to >16	0.5	0.2	99.3
Piperacillin Tazobactam	1070	>64	>64	≤2 to >64	1.0	0.6	98.4
**CR-*A. baumannii*, RTI**							
Colistin	756	≤1	≤1	≤1 to >8	95.4	0	4.6
Amikacin	756	>32	>32	≤4 to >32	18.0	0.5	81.5
Cefepime	756	>32	>32	2 to >32	1.6	2.7	95.8
Ciprofloxacin	756	>2	>2	≤0.25 to >2	2.1	0.8	97.1
Imipenem	756	32	>32	1 to >32	0.4	0.1	99.5
Meropenem^∗^	641	>16	>16	1 to >16	0.6	0.3	99.1
Piperacillin Tazobactam	756	>64	>64	≤2 to >64	1.1	0.7	98.3
**CR-*A. baumannii*, IAI**							
Colistin	265	≤1	≤1	≤1 to >8	98.1	0	1.9
Amikacin	265	>32	>32	≤ 4 to >32	14.3	1.9	83.8
Cefepime	265	>32	>32	4 to >32	0.4	0.8	98.9
Ciprofloxacin	265	>2	>2	≤0.25 to >2	0.8	0	99.3
Imipenem	265	32	>32	2 to >32	0.4	0	99.6
Meropenem^∗^	168	>16	>16	8 to >16	0	0	100.0
Piperacillin Tazobactam	265	>64	>64	16 to >64	0.8	0.4	98.9
**CR-*A. baumannii*, UTI**							
Colistin	45	≤1	≤1	≤1 to >4	95.6	0	4.4
Amikacin	45	>32	>32	≤4 to >32	6.7	0	93.3
Cefepime	45	>32	>32	4 to >32	2.2	0	97.8
Ciprofloxacin	45	>2	>2	>2 to >2	0	0	100.0
Imipenem	45	>32	>32	8 to >32	0	0	100.0
Meropenem^∗^	35	>16	>16	8 to >16	0	0	100.0
Piperacillin Tazobactam	45	>64	>64	16 to >64	2.2	0	97.8
**MDR-*A. baumannii*, all sources**							
Colistin	1090	≤1	≤1	≤1 to >8	95.7	0	4.3
Amikacin	1090	>32	>32	≤4 to >32	16.7	0.8	82.5
Cefepime	1090	>32	>32	2 to >32	1.0	2.4	96.6
Ciprofloxacin	1090	>2	>2	≤0.25 to >2	1.1	0.6	98.3
Imipenem	1090	32	>32	≤0.5 to >32	2.8	0.3	97.0
Meropenem^∗^	860	>16	>16	≤0.12 to >16	2.3	0.7	97.0
Piperacillin Tazobactam	1090	>64	>64	≤2 to >64	1.3	0.5	98.3
**MDR-*A. baumannii*, RTI**							
Colistin	765	≤1	≤1	≤1 to >8	95.4	0	4.6
Amikacin	765	>32	>32	≤4 to >32	17.5	0.5	82.0
Cefepime	765	>32	>32	2 to >32	1.1	3.0	96.0
Ciprofloxacin	765	>2	>2	≤0.25 to >2	1.2	0.8	98.0
Imipenem	765	32	>32	≤0.5 to >32	2.4	0.3	97.4
Meropenem^∗^	649	>16	>16	≤0.12 to >16	2.2	0.8	97.1
Piperacillin Tazobactam	765	>64	>64	≤2 to >64	0.9	0.5	98.6
**MDR-*A. baumannii*, IAI**							
Colistin	270	≤1	≤1	≤1 to >8	97.0	0	3.0
Amikacin	270	>32	>32	≤4 to >32	14.8	1.9	83.3
Cefepime	270	>32	>32	8 to >32	0.4	1.1	98.5
Ciprofloxacin	270	>2	>2	≤0.25 to >2	0.4	0	99.6
Imipenem	270	32	>32	≤0.5 to >32	2.2	0.4	97.4
Meropenem^∗^	172	>16	>16	≤0.12 to >16	2.3	0.6	97.1
Piperacillin Tazobactam	270	>64	>64	≤2 to >64	1.1	0.4	98.5
**MDR-*A. baumannii*, UTI**							
Colistin	51	≤1	≤1	≤1 to >4	92.2	0	7.8
Amikacin	51	>32	>32	≤4 to >32	9.8	0	90.2
Cefepime	51	>32	>32	4 to >32	3.9	0	96.1
Ciprofloxacin	51	>2	>2	1 to >2	2.0	2.0	96.1
Imipenem	51	32	>32	≤0.5 to >32	11.8	0	88.2
Meropenem^∗^	37	>16	>16	≤0.12 to >16	5.4	0	94.6
Piperacillin Tazobactam	51	>64	>64	4 to >64	7.8	0	92.2

*^∗^There was no meropenem tested in 2015. The data are expressed as n (%). MIC breakpoint (mg/L) of colistin to *A. baumannii* for susceptibility is ≤2; resistance is >2 according to Clinical and Laboratory Standards Institute [CLSI] (2017) Performance Standards for Antimicrobial Susceptibility Testing, 27th Edition, 2017.*

Colistin (94.1 and 90.7%) had the highest susceptibility rates against CR and MDR *P. aeruginosa*, followed by amikacin (73.4 and 68.7%). Colistin susceptibility rates of CR and MDR *P. aeruginosa* isolates were 95.6 and 91.79% for respiratory tract, 89.7 and 87.2% for intra-abdominal as well as 92.5 and 89.6% for urinary tract infections. Susceptibilities of CR *P. aeruginosa* to cefepime and ciprofloxacin were 32.7% for RTIs and in the range of 46.6 – 53.5% for IAIs and 20 – 27.5% for UTIs, whereas for MDR and CR *P. aeruginosa* RTI, IAI and UTI isolates susceptibilities to the other tested antibiotics were ≤35% (Table 4).

**TABLE 4:** *In vitro* activity of 7 antimicrobial agents against CR and MDR isolates of *P. aeruginosa* collected in China from 2015 to 2017.

**Organism/specimen source/antimicrobial agent**	***N***	**MIC (mg/L)**			**MIC interpretation**		
		**MIC_50_**	**MIC_90_**	**MIC range**	**Susceptible (%)**	**Intermediate (%)**	**Resistant (%)**
**CR-*P. aeruginosa*, all sources**							
Colistin	522	≤1	2	≤1 to >8	94.1	0	5.9
Amikacin	522	≤4	>32	≤4 to >32	73.4	1.5	25.1
Cefepime	522	16	>32	≤1 to >32	35.4	16.3	48.3
Ciprofloxacin	522	2	>2	≤0.25 to >2	36.2	8.1	55.8
Imipenem	522	16	>32	1 to >32	1.3	2.3	96.4
Meropenem^∗^	418	16	>16	≤0.12 to >16	4.3	10.1	85.7
Piperacillin Tazobactam	522	>64	>64	≤2 to >64	25.1	15.9	59.0
**CR-*P. aeruginosa*, RTI**							
Colistin	361	≤1	2	≤1 to >4	95.6	0	4.4
Amikacin	361	≤4	>32	≤4 to >32	73.4	1.4	25.2
Cefepime	361	32	>32	≤1 to >32	32.7	17.2	50.1
Ciprofloxacin	361	>2	>2	≤0.25 to >2	32.7	8.9	58.5
Imipenem	361	16	>32	1 to >32	0.8	2.8	96.4
Meropenem^∗^	303	16	>16	0.25 to >16	3.0	8.6	88.5
Piperacillin Tazobactam	361	>64	>64	≤2 to >64	22.7	15.5	61.8
**CR-*P. aeruginosa*, IAI**							
Colistin	116	≤1	4	≤1 to >8	89.7	0	10.3
Amikacin	116	≤4	>32	≤ 4 to >32	75.9	1.7	22.4
Cefepime	116	16	>32	≤1 to >32	46.6	10.3	43.1
Ciprofloxacin	116	0.5	>2	≤0.25 to >2	53.5	6.0	40.5
Imipenem	116	16	>32	1 to >32	1.7	0	98.3
Meropenem^∗^	74	8	>16	≤0.12 to >16	8.1	17.6	74.3
Piperacillin Tazobactam	116	64	>64	≤2 to >64	36.2	13.8	50.0
**CR-*P. aeruginosa*, UTI**							
Colistin	40	≤1	2	≤1 to >4	92.5	0	7.5
Amikacin	40	≤4	>32	≤4 to >32	65.0	2.5	32.5
Cefepime	40	16	>32	≤1 to >32	27.5	25.0	47.5
Ciprofloxacin	40	>2	>2	≤0.25 to >2	20.0	7.5	72.5
Imipenem	40	16	>32	1 to >32	5.0	5.0	90.0
Meropenem^∗^	36	16	>16	1 to >16	8.3	5.6	86.1
Piperacillin Tazobactam	40	>64	>64	4 to >64	15.0	22.5	62.5
**MDR-*P. aeruginosa*, all sources**							
Colistin	486	≤1	2	≤1 to >8	90.7	0	9.3
Amikacin	486	8	>32	≤4 to >32	68.7	2.1	29.2
Cefepime	486	32	>32	≤1 to >32	11.1	19.6	69.3
Ciprofloxacin	486	> 2	>2	≤0.25 to >2	28.8	6.6	64.6
Imipenem	486	16	>32	≤0.5 to >32	23.1	5.1	71.8
Meropenem^∗^	398	16	>16	≤0.12 to >16	24.4	4.5	71.1
Piperacillin Tazobactam	486	>64	>64	≤2 to >64	6.4	12.1	81.5
**MDR-*P. aeruginosa*, RTI**							
Colistin	341	≤1	2	≤1 to >8	91.8	0	8.2
Amikacin	341	8	>32	≤4 to >32	68.3	2.1	29.6
Cefepime	341	32	>32	≤1 to >32	10.6	19.4	70.1
Ciprofloxacin	341	>2	>2	≤0.25 to >2	25.5	7.9	66.6
Imipenem	341	16	>32	≤0.5 to >32	20.5	6.5	73.0
Meropenem^∗^	289	16	>16	≤0.12 to >16	21.8	4.8	73.4
Piperacillin Tazobactam	341	>64	>64	≤2 to >64	5.3	11.7	83.0
**MDR-*P. aeruginosa*, IAI**							
Colistin	94	≤1	>4	≤1 to >8	87.2	0	12.8
Amikacin	94	8	>32	≤4 to >32	69.2	2.1	28.7
Cefepime	94	>32	>32	≤1 to >32	7.5	19.2	73.4
Ciprofloxacin	94	2	>2	≤0.25 to >2	43.6	2.1	54.3
Imipenem	94	16	>32	≤0.5 to >32	27.7	1.1	71.3
Meropenem^∗^	62	8	>16	≤0.12 to >16	30.7	4.8	64.5
Piperacillin Tazobactam	94	>64	>64	≤2 to >64	9.6	8.5	81.9
**MDR-*P. aeruginosa*, UTI**							
Colistin	48	≤1	4	≤1 to >4	89.6	0	10.4
Amikacin	48	≤4	>32	≤4 to >32	70.8	2.1	27.1
Cefepime	48	32	>32	4 to >32	22.9	20.8	56.3
Ciprofloxacin	48	>2	>2	≤0.25 to >2	25.0	6.3	68.8
Imipenem	48	8	>32	≤0.5 to >32	33.3	4.2	62.5
Meropenem^∗^	44	16	>16	≤0.12 to >16	34.1	2.3	63.6
Piperacillin Tazobactam	48	>64	>64	4 to >64	8.3	20.8	70.8

*^∗^Meropenem was not examined in 2015, only after 2016. The data are expressed as n (%). MIC breakpoint (mg/L) of colistin to *P. aeruginosa* for susceptibility is ≤2; resistance is >2 according to Clinical and Laboratory Standards Institute [CLSI] (2017) Performance Standards for Antimicrobial Susceptibility Testing, 27th Edition, 2017.*

### Difference in Sensitivity of *P. aeruginosa* and *A. baumannii* to Colistin in Different Departments

**FIGURE 2:**
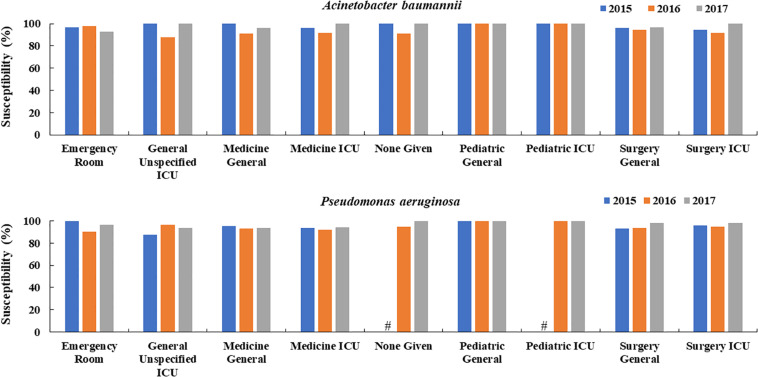
Changes in the susceptibility of colistin to *A. baumannii* and *P. aeruginosa* over time in different hospital departments in China (2015, 2016, 2017). ^#^Means the susceptibility data were not available.

Overall, sensitivity rates of *P. aeruginosa* and *A. baumannii* to colistin were >85% in all departments. There was no pattern of increased resistance rates over the study period in any department and the susceptibilities in 2017 were mostly higher or equal to the rates in 2015 and 2016 in all included departments (Figure 2).

### Difference in Sensitivity of *P. aeruginosa* and *A. baumannii* to Colistin in Different Regions

**FIGURE 3:**
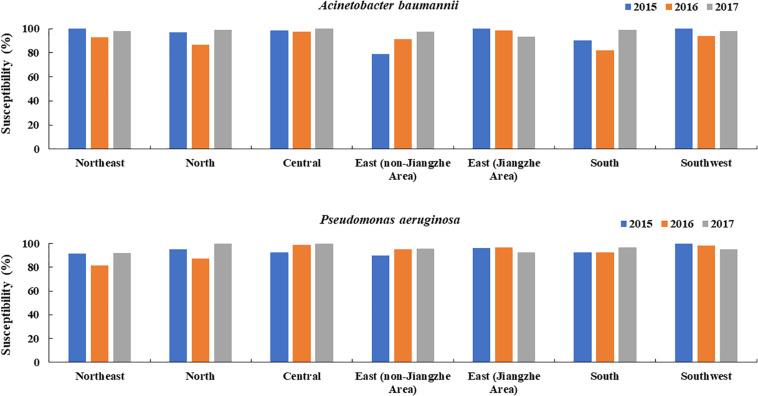
Changes in the susceptibility of colistin *to A. baumannii* and *P. aeruginosa* over time in different regions of China (2015, 2016, 2017).

Similarly to departments, in all regions of China tested, *A. baumannii* and *P. aeruginosa* sensitivities to colistin were constantly above 80% and apart from a slight tendency of *P. aeruginosa* and *A. baumannii* in east Jiangzhe there was no visible sensitivity decrease over the years in all other regions (Figure 3).

## Discussion

MDR has become a worldwide problem (WHO, 2014). Since the rise of extended spectrum β-lactamase-positive *Enterobacteriaceae*, carbapenems have served as the gold standard for empirical treatment of ESBL-producing *Enterobacteriaceae* infections over decades (Papp-Wallace et al., 2011; Vardakas et al., 2012; Tamma et al., 2015). However, since carbapemase-producing *Enterobacteriaceae* have become increasingly prevalent in the last decade, susceptibility rates, particularly for hospital acquired infections, dropped to levels which urge the need for alternative treatments (Routsi et al., 2013; Zurawski, 2014; Sheu et al., 2018).

In recent years, *A. baumannii* isolates have developed resistances to carbapenems, amikacin, ciprofloxacin, cefepime and piperacillin-tazobactam (Labarca et al., 2016; Ren et al., 2019). It is noteworthy that CR *A. baumannii* and CR *P. aeruginosa* are listed as priority pathogens posing the greatest threat to human health by the WHO (Willyard, 2017). In a survey including 312,075 Gram-negative isolates collected in the US in 2017, *A. baumannii* and *P. aeruginosa* comprised the most common CR isolates mainly from ICU settings, with rates of 36.6 and 14.6% (Mccann et al., 2018), which was essentially lower than CR rates of 78.7% for *A. baumannii* and 38.9% for *P. aeruginosa* in the present study, indicating that the situation in China is by far more dramatic compared to the US. However, in our study *P. aeruginosa* and *A. baumannii* isolates were predominantly obtained from RTIs in which the CR and MDR rates of both strains were essentially highest, in good agreement with previously published findings (Mccann et al., 2018). These results may be attributed to the fact that *P. aeruginosa* and *A. baumannii* predominantly cause ventilator-associated pneumonia especially in critically ill patients in ICUs in which CR and MDR rates are generally high (Shete et al., 2010; Tao et al., 2011; Ding et al., 2016; Borgatta et al., 2017; Gupta et al., 2017; Du et al., 2019). However, airborne transmission has also been postulated for the transmission of *A. baumannii* pneumonia (Whitman et al., 2008; Munoz-Price et al., 2013; Spellberg and Bonomo, 2013).

Susceptibilities of *A. baumannii* isolates to amikacin, ciprofloxacin, cefepime and piperacillin-tazobactam were only in the range 19.2 – 33.0% and for *P. aeruginosa* 55.6 – 63.1% to ciprofloxacin, cefepime and piperacillin-tazobactam, but 83.4% to amikacin, indicating that for all tested antimicrobial agents other than colistin, at best only amikacin might serve as an empiric treatment option for *P. aeruginosa* infections. In contrast, overall susceptibility rates to colistin were 94.6 and 95.7% for *P. aeruginosa* and *A. baumannii* in isolates obtained from IAIs, UTIs and RTIs in the present study, which is in agreement with previously reported high susceptibility rates of both species in other countries (Ece et al., 2014; Malekzadegan et al., 2019). In addition, there was no visible trend of decreasing colistin susceptibilities toward 2017 in the different hospital departments and Chinese regions, except for a slight decrease in east Jiangzhe. The constant low resistant rates might be explained by the fact that colistin has been mainly used for human clinical medication in China since 2017 (WHO, 2018) and earlier colistin resistance of *Enterobacteriaceae* in China has been attributed to veterinary use (Chen et al., 2011; Yang et al., 2017; Wang et al., 2018). The resistance mechanism to colistin of these small number of isolates may be associated with chromosomal mutations (Dafopoulou et al., 2015) or defective biofilm formation (Azimi and Lari, 2019).

### Limitations

The present study presents only data from 2015 to 2017, because the SMART surveillance program is retrospective. In addition, we reported the susceptibility of *A. baumannii* and *P. aeruginosa* to 7 commonly used antibiotics in China. The susceptibilities of other non-fermenting bacteria such as *Stenotrophomonas, Burkholderia* were not included due to the limited number of strains.

## Conclusion

Due to high MDR and CR rates of *A. baumannii* (80.1 and 78.7%) and *P. aeruginosa* (36.2 and 38.9%) isolates from RTIs, IAIs and UTIs, between 2015 and 2017 high susceptibility rates were only detected for colistin.

## Data Availability Statement

The SMART database is not public and only accessible for SMART investigators, but the data that support the findings of this study are directly available from MSD China or from the authors upon reasonable request and with permission of MSD China.

## Ethics Statement

The protocol has been reviewed by the Human Research Ethics Committee of the Institutional Review Board (IRB) of the Peking Union Medical College Hospital and since the project falls under the category observational study and all bacterial strains were from residual samples used in clinical diagnosis or were strains from their subcultures, it met the criteria for exemption. This project did not involve any patient information nor did it affect the normal diagnosis and treatment of patients, and after consultation with the IRB, formal ethical approval was reviewed and waived; written patient consent was not required (Ethics Approval Number: S-K238).

## Author Contributions

All the authors read and approved the submitted manuscript and solely responsible for the conception and implementation of the study and for writing the manuscript. HZ, QY, and YX conceptualized the study. HZ, GZ, JZ, SD, YK, QY, and YX collected the data. HZ, QY, and YX analyzed the data. HZ wrote the original draft. HZ, GZ, JZ, SD, YK, QY, and YX wrote, reviewed, and edited the manuscript.

## Conflict of Interest

YK was employed by MSD China.

The remaining authors declare that the research was conducted in the absence of any commercial or financial relationships that could be construed as a potential conflict of interest.
